# Supplementary material for: Trauma-informed care for everyone: A study on patient and provider perspectives in an urban primary care setting
Source: PLoS One. 2026 May 15;21(5):e0349308. doi: 10.1371/journal.pone.0349308 (PMC13178877; doi:10.1371/journal.pone.0349308)
Supplement: Attachment — Submitted filename: Peer Review Responses PONE-D-25-19371_27-Feb-2026.docx [file pone.0349308.s002.docx]

**Response to Reviewers Manuscript PONE-D-25-19371**

We are grateful for the thoughtful suggestions provided by the editorial team and peer reviewers, which have helped strengthen our manuscript. Please see a summary of the comments and our responses in the table below.

**Table: Summary of comments, revisions, and edits**

| **Comment/Feedback** | **Revisions/Comments** |
| --- | --- |
| **Journal requirements** | |
| 1. Manuscript formatting in line with PLOS ONE style. 2. Grant information does not match. 3. State role of funders in this research 4. Remove funding-related text in manuscript 5. Third party data 6. Citation recommendations | 1. Done 2. Thank you for highlighting this. We have revised the funding information in the editorial manager system to be aligned with the funding statement. The grant information/statement should now state:   *This study was supported by the Innovation Fund of the Alternative Funding Plan for the Academic Health Sciences Centres of Ontario (Grant number: SMH 20-006). The funders had no role in study design, data collection and analysis, decision to publish, or preparation of the manuscript.*   3. Funders had no role in the study design, data collection and analysis, decision to publish, or preparation of the manuscript. We have added the suggested sentence in the cover letter as outlined. 4. We have removed the funding statement information in the manuscript text. 5. This research does not involve any third-party data. We have updated the data availability statement (as proposed by the peer reviewer) to be more concise, as follows:   *Supporting study data are not publicly available. According to participant study letters of information and ethics consent forms data cannot be shared outside of the research team. Sharing data could compromise participant trust, confidentiality, and privacy. For questions or data access requests please contact the Research Ethics Office at Unity Health Toronto* [*researchethics@unityhealth.to*](mailto:researchethics@unityhealth.to)*.*   6. Noted. |
| **Reviewer 1** | |
| This is a well-conceived and timely qualitative manuscript that addresses an important and underexplored area: how to practically implement trauma informed care (TIC) in an interdisciplinary urban primary care setting, from the perspectives of both patients and providers. The topic is highly relevant to primary care, mental health, and health equity, and the paper contributes useful, practice-oriented insights that can inform clinical programs and policy discussions.  The manuscript’s strengths are rooted in its patient-oriented and interpretative qualitative methodology, which ensures that the findings are grounded in the lived experiences of both those receiving and delivering care. By interviewing 10 patients and 13 providers, the authors successfully triangulate perspectives to highlight the bidirectional nature of trust and the shared challenges of implementing trauma-informed care (TIC). The research is further bolstered by the active engagement of three patient advisors with lived experience, who guided the study's design and interpretation. Ethically, the study is sound, maintaining a high standard of transparency regarding its research ethics board approval and providing a clear, consent-based justification for restricted data access to protect participant confidentiality. Finally, the manuscript effectively situates its findings within robust literature, demonstrating a clear understanding of how TIC serves as a foundational framework for health equity and holistic primary care. | Thank you for this thoughtful comment and reflection on the study strengths and contributions to scholarship. |
| - While the study identifies systemic barriers like billing and time constraints, the manuscript would benefit from more concrete "low-barrier" institutional suggestions (e.g., specific intake form changes) that clinics can implement immediately. - The patient sample was predominantly born in Canada and had completed post-secondary education. This lacks perspectives from newcomers, those who do not speak English as a first language, and individuals with different educational backgrounds. - While the study recommends a "whole team" approach to TIC, the data collection was restricted to primary care providers (physicians and nurse practitioners). It excludes the vital perspectives of administrative staff, social workers, and allied health professionals. - The research was conducted exclusively in a large, multi-site urban family health team in Toronto. These findings may not be generalizable to the specific resource constraints or patient contexts found in rural or remote settings. - The study identifies deep-seated systemic barriers such as billing models, short appointment times, and a lack of public mental health resources that the intervention itself cannot resolve without broader policy intervention. | - Thank you for this comment. We agree and have integrated tangible examples of recommendations (as outlined below). - We have incorporated this feedback in the discussion section on study limitations outlining gaps in recruitment efforts and avenues for future research (p.25) - This is an excellent point, and we have incorporated gaps in recruitment of allied health professionals as a study limitation and underscored the importance of examining their experiences in future research to better understand clinic/systemic prospects and challenges to implementing TIC as a “whole team”. - We agree, this gap was outlined as a study limitation in the original manuscript. No edits made. - No changes recommended/made based on this observation. |
| **Discussion**   - Expand the Discussion to address limitations regarding newcomers and non-English speakers. - Discuss how excluding non-clinical staff (clerical/allied health) from the sample effects "whole team" conclusions. - Provide concrete examples of immediate clinic-level changes (e.g., intake forms, environment). - Clarify the boundary between primary care universal precautions and specialized trauma services. | - Thank you for these helpful recommendations. We have incorporated these suggestions as study limitations (p.25). - We agree and have acknowledged the exclusion of non-clinical staff as participants as an important gap/limitation of this research and possible avenue for future work related to trauma-informed care (p.25). - We have outlined concrete examples of clinic-level recommendations, including changes to intake procedures/forms (p.21). - Thank you for this suggestion. We have clarified the distinct domains/descriptions of *universal trauma precautions and trauma-specific care/strategies* (including examples as outlined by Raja et al (2015)) (see discussion section, p.22). These edits also address comments from Reviewer #3. |
| **Reviewer 2** | |
| **Clarification of Transferability:** While the study context is clearly described, the manuscript would benefit from a brief, explicit discussion on how findings may (or may not) transfer to other primary care settings (e.g., rural clinics, non-academic practices, or non-Canadian health systems). | We appreciate this feedback. We have added some text in the data analysis which highlight possibilities for generalizing or applying our findings to other contexts. |
| **Operational Definition of Trauma-Informed Care:** Although TIC is well discussed conceptually, a concise operational definition early in the manuscript (e.g., in the Introduction or Methods) would help readers clearly distinguish TIC from overlapping frameworks such as patient-centred or equity-oriented care. | Thank you for this suggestion. We have more clearly communicated our understanding/conceptualization of TIC in this research in the introduction (p.3). The operational definition used includes:  *In this study, we conceptualize TIC as principles or guidelines for routine patient primary health care that promotes patient trust, respect, safety, collaboration, and empowerment* (1–3)*.* |
| **Participant Characteristics:** Consider providing a summary table of participant demographics (patients and providers separately). This would improve transparency and allow readers to better contextualize the findings. | We really value this feedback and have integrated additional detail (including related to ethnicity and age) in the narrative description of study participants to help guide and contextualize interpretation of results (p.8). Institutional policy at the Department of Community and Family Medicine does not allow the study team to publish research with participant data (with cell sizes <5) due to concerns related to anonymity. Therefore, we have also added the rationale/justification for exclusion of the demographic table (p.8). |
| **Consistency in Terminology:** Ensure consistent use of terms such as trauma-informed care, trauma-informed primary health care, and TIPHC throughout the manuscript. | Thank you for highlighting this. We have removed the use of TIPHC throughout. We have largely used “trauma-informed care” within participant quotes or the acronym (TIC) throughout. |
| **Data Availability Statement:** The rationale for restricted data access is appropriate; however, the statement could be slightly streamlined for clarity and conciseness. | Done. |
| **Editing and Style**: Minor grammatical and stylistic edits would further improve readability (e.g., sentence length in the Discussion section). | We have made substantial edits to the discussion to increase readability and reduce sentence length. |
| **Reviewer 3** | |
| 1. Please explain - How were patients recruited (posters, clinician referral, purposive sampling, convenience)? How were providers recruited? Who approached whom? Any incentives? | Thank you for this comment. We have provided additional detail regarding our sampling and recruitment strategies (p.5).  We have also outlined that all participants received honoraria for their involvement in this study. |
| 2. Why 10 patients and 13 providers was “enough” for the aims (Please explain information power, saturation logic, pragmatic constraints). | We initially aimed to recruit 10-25 patients and 10-25 health care providers in this study. Sampling and recruitment plans were informed by logistical and pragmatic factors (e.g., grant funding timelines and specific eligibility criteria of patients and primary care providers at the family health team). After 12+ months of recruitment, we successfully recruited 10 patients and 13 providers. With discussions averaging 40 minutes, interviews offered extremely rich and insightful discussions about their knowledge/experiences related to TIC at the SMAFHT.  Qualitative scholars have increasingly suggested that ‘data saturation’ is more aligned with/reflective of positivist qualitative orientations/paradigms. The concept of (data) saturation or “information redundancy or the point at which no new themes of codes ‘emerge’ from data” is often “taken for granted” and uncritically employed to justify sample sizes in qualitative research (Braun & Clarke, 2021, p.201). Several authors (please see works by Braun & Clarke (4), Malterud et al. (5), and Reilly and Parker (6)) argue that using saturation as a measure of quality or determining sample size is “inappropriate” (O’Reilly & Parker, 2013, p.190). Given this rationale, we have not incorporated concepts of power or data saturation in the manuscript as they are incongruent with the interpretive epistemological perspective guiding this research.  However, we have outlined that the use of “information power” as proposed by Malterud et al. may be a useful concept to understand quality and sufficiency of qualitative data sample sizes (5). We offer a justification of applying this concept due to the specific characteristics/eligibility criteria for this research (e.g., patients and providers at a family health team), purposive sampling procedures, use of a interpretive theoretical approach, the depth/quality of interview dialogue (e.g., interviews were very rich in detail), and applying robust analytic strategies (p. 7). |
| 3. Provide a participant table (PLOS ONE-friendly) with key demographics for both groups (age bands, gender, race/ethno-racial identity, role type; for patients: housing stability or self-rated financial strain if collected). | We really value this feedback and have integrated additional detail (including related to ethnicity and age) in the narrative description of study participants to help guide and contextualize interpretation of results (p.8).  Internal research policy at the Department of Family and Community Medicine at St Michael’s Hospital does not  allow publication of participant data with cell sizes <6 due to concerns related to anonymity. We have also described the rationale for excluding a demographic table within the manuscript for transparency and to offer further context for readers (p.8). |
| 4. Patients had to “self-identify as having experienced a traumatic event.” That excludes: people with trauma who do not label it as trauma, those with dissociation/avoidance, shame, low trust, language barriers, patients harmed by the system who avoid engagement (arguably central to TIC). Name this explicitly as a selection mechanism and clarify in Limitations that findings likely reflect perspectives of patients already able/willing to disclose and engage. | Thank you for highlighting this, we have extended our consideration of potential study limitations related to participant self-identification/selection, including individuals who may not self-identify as having experienced trauma or those with low trust in the health system (p.25-26). |
| 5. The study states reflexive thematic analysis, in vivo coding, NVivo, and collaborative review. Please state:  - Was coding inductive, deductive, or hybrid (you imply hybrid: literature + in vivo)? Specify explicitly.  - How were themes generated (semantic vs latent)?  - How was disagreement handled (not necessarily “inter-rater reliability,” but describe consensus building).  - Were patient partners involved in interpretation beyond “feedback on design and interpretation”? How, exactly?  - Add a short analytic workflow bullet list: 1) familiarization, 2) initial coding, 3) theme candidate generation, 4) review/refinement, 5) naming/definition, 6) write-up with exemplar quotes.  Include an explicit statement on information power/saturation or why saturation is not the goal in reflexive thematic analysis, but adequacy was achieved. | - We have clarified that we employed abductive analytic reasoning – drawing inductively from transcript data and more deductively (e.g., literature and interview guides) to inform knowledge production (p.6). - Recent scholarship by Braun & Clarke (see Braun & Clarke’s 2019 article ‘reflecting on reflexive thematic analysis’ (7)) offers a critique of adopting dichotomous or rigid choices related to semantic *vs* latent coding/thematic analysis. Suggesting that this is a “misapplication” of their analytic method. Therefore, we have underscored that in this research ‘themes’ represent analytic outputs, creative/interpretive stories and “patterns of shared meaning underpinned or united by a core concept” (Braun & Clarke, 2019, p.593) (p.6-7). - Data analysis (and any disagreements in analysis) were informed by a “collaborative and reflexive” approach to generate a more nuanced and complex understanding of the data rather than seeking consensus or specific ‘truth’ (Braun & Clarke, p.594). We have clarified this process on pages 6-7. - We have provided additional detail to outline the role/contributions of patient advisors in the research process, including analysis and interpretation (p.7). - We have integrated numbered bullet points and added further detail to highlight the analytic process. However, we have also emphasized that this represents a “fluid and recursive” process as opposed to rigid/linear phases as underscored by Braun & Clarke (2019) (p.6-7). - As highlighted above, given the debates in qualitative scholarship related to power/data saturation we have drawn from Malterud (2016) and Tracy (2010) to guide (and illustrate) rigour of this work (p.7). |
| **6. The introduction discusses feasibility/acceptability of universal screening and concerns about harm.**  In Results, participants disagree about how directly to ask about trauma, but the manuscript does not clearly delineate: universal trauma precautions (assume trauma history, focus on safety/choice/agency), versus routine trauma screening (asking about trauma exposure/ACEs).   - Make this distinction explicit in Discussion using the two-domain framing you cite (Raja et al.). - Consider adding a subtheme or short section: “Approaches to asking about trauma: universal precautions vs screening” with clear clinical implications (when/why each might be appropriate). | - We appreciate this feedback and have made edits to address similar comments from Reviewer 1 to more clearly define universal trauma precautions and trauma-specific care (p.22). - Thank you for this suggestion, we have divided the subtheme “TIC strategies/approaches employed by primary health care providers” into two sub-themes to address this feedback (e.g., “TIC strategies employed by primary care providers” and “Approaches to asking about trauma”). In this sub-theme, we examine patient participants recommendations related to screening/asking about their history of trauma. Clinical implications and recommendations are synthesized in the discussion section. |
| 7. References:  - The discussion includes “(Roberts et al., 2019)” in text rather than numbered citation format used elsewhere. This is inconsistent and may indicate a missing/incorrectly linked reference in the numbered list. - Felitti (2002) entry contains a typo (“adult healht”). - Hopper et al. (2010) citation appears to include artifact text (“~!2009-08-20~!...”). - CIHR (2014) reference looks incomplete/odd (“NeHC \| HIMSS. 19.”). Likely not in proper format; verify the correct source and include URL/access date if web-based. - Unity Health Toronto (2024) reference is a web page; ensure access date and full URL stability.  - If the manuscript discusses universal screening feasibility and potential harms, consider whether the cited sources sufficiently cover the debate (you cite Finkelhor cautions on ACE screening, and concerns about negative impacts). It would help to ensure at least one primary care ACE screening evidence review is cited (if already in manuscript, confirm it’s correctly formatted).  8. Please add following limitations. - All patient participants had at least some post-secondary education. This likely underrepresents those facing greatest structural barriers, potentially inflating acceptability of TIC conversations and advocacy capacity.  - Interviews were via Zoom Healthcare. That may exclude patients with limited privacy, limited internet, unstable housing, or discomfort with video calls and may change disclosure patterns.  - The manuscript notes the importance of Indigenous, Black, LGBTQ+ representation, but does not report participant composition on these dimensions. If the sample is not diverse, the paper should acknowledge limits in making equity-oriented conclusions.  - Participants often equate TIC with “treating a human being well,” overlapping with patient-centred care. Without careful framing, the study risks definitional dilution: findings may reflect general good communication rather than TIC-specific components (e.g., power dynamics, re-traumatization mitigation, staff support systems).  - The results emphasize positive TIPHC framing; please include whether you actively looked for disconfirming evidence (e.g., patients who prefer not to discuss trauma, or providers who reject screening). | - Thank you for this review of the reference list. We have made all edits as suggested. - We can confirm the access date is provided and the URL remains active. - Thank you for this recommendation. We have added further citations to describe debates/concerns related to universal trauma screening vs precautions in general (e.g., Raja et al (8,9)) and specific citations to outline concerns/challenges to universal screening and implementation of TIC strategies in primary care settings, more specifically (10,11). We have also more clearly synthesized the potential challenges/impacts of universal screening in the introduction (p.3-4). - We have added educational background as one of the potential study considerations and areas for future research. - Thank you for this feedback. We have integrated this as a potential limitation, yet also acknowledge that virtual video interviewing may have simultaneously increased accessibility of participating in this research for some groups (12). - As highlighted above, we did not include a participant demographic table due to internal research policies related to publication of participant data with small cell sizes (e.g., cell sizes <6). However, we have suggested that increased effort to diverse groups experience health inequities is necessary to more clearly reflect and understand experiences of TIC. - This study is informed by participants’ lived experiences of accessing or providing trauma-informed care in a primary care setting. Interview questions explored patient and provider understanding of trauma-informed care and their experiences of accessing/providing this type of care. Participants suggested that trauma-informed care and patient-centred approaches are complementary and interrelated. Similarly, literature (for example: Raja et al (2015 & 2019) suggest that patient-centred care is foundational to delivering trauma-informed care and that employing patient-centred communication and behavioural techniques is helpful to reduce patient anxiety and increase physician rapport, without requiring trauma screening or specific knowledge of patients’ trauma history. Therefore, our findings are aligned with existing literature and models of trauma-informed care. We have edited some of the language for clarity to highlight that TIC and patient-centred care represent distinct, yet complementary concepts (p.10). - Thank for you this comment. All 10 patient participants emphasized that it was important to them that their health care providers had knowledge of their history of trauma. However, perspectives were mixed on how providers should ask/screen for trauma (with several suggesting integration into medical history conversations would be acceptable/appropriate). A few participants suggested that leaving “space” or the “door open” for patients to share about their history would be preferable for them. Several participants emphasized that using open-ended questions and creating routine opportunities to “check-in” could be employed. However, no patient participants suggested that trauma should be avoided in conversations with their primary care providers or mentioned that they would prefer not to discuss trauma with their primary care providers. We have clarified some of the nuances of these findings (p.14).   Similarly, all health care providers suggested that trauma-informed care was important to their primary care practices. This was demonstrate amidst varied experiences/knowledge with TIC, with those who were less familiar suggesting it was important (and that they wanted further information on how to practice TIC). |
| **Reviewer 4** | |
| Title: Make it more relevant to the study, to attract readers. | Thank you, we revised the title of this article to more clearly attract readers. |
| Introduction: Better streamlining is needed. No clear objectives were mentioned, although knowledge was addressed to some extent. Minimize the redundancy of the statements. | We have more clearly communicated the research objectives. |
| Methods: Well-detailed. Polish the data analysis. | We have provided further detail on the data analysis strategies employed, including reflexing thematic analysis (p.6-7). |
| Results: Well-described and organized. Improve the clarity and minimize the repetitions. | Thank you for this observation. We appreciate the positive feedback on manuscript flow/organization. The suggestion to improve clarity and minimize repetition is quite broad. We have reviewed/edited the results section carefully to avoid redundancy. We would be happy to address specific areas of concern if further information is additional feedback is provided. |
| Limitations: Again, improve the clarity, and address the steps taken to minimize or negate the limitations. | We have edited the research limitations section and incorporated feedback from other peer reviewers (p.25-26). |
| Conclusions: Mention future research guidance.  Overall, readability is reasonable, but provide clearer and concise statements. Minimize the redundancy. | We have more clearly emphasized areas for future research, including research that examines experiences of allied health professionals and other clinic staff to implement TIC (e.g., a “whole team approach”).  In addition, we have highlighted gaps in study sampling and acknowledge that increased representation of groups that experience health inequities would help advance understanding of possible structural barriers to TIC (and primary care, more broadly) (p.25-26). |

**References**

1.	Palmieri J, Valentine JL. Using Trauma-Informed Care to Address Sexual Assault and Intimate Partner Violence in Primary Care. Journal for Nurse Practitioners. 2021;17(1):44–8. doi:10.1016/j.nurpra.2020.08.028

2.	Kokokyi S, Klest B, Anstey H. A patient-oriented research approach to assessing patients’ and primary care physicians’ opinions on trauma-informed care. PLoS ONE. 2021;16(7 July):1–20. doi:10.1371/journal.pone.0254266

3.	Elliott DE, Bjelajac P, Fallot RD, Markoff LS, Reed BG. Trauma-informed or trauma-denied: Principles and implementation of trauma-informed services for women. Journal of Community Psychology. 2005;33(4):461–77. doi:10.1002/jcop.20063

4.	Braun V, Clarke V. To saturate or not to saturate? Questioning data saturation as a useful concept for thematic analysis and sample-size rationales. Qualitative Research in Sport, Exercise and Health. 2021;13(2):201–16. doi:10.1080/2159676X.2019.1704846

5.	Malterud K, Siersma VD, Guassora AD. Sample Size in Qualitative Interview Studies: Guided by Information Power. Qual Health Res. 2016 Nov;26(13):1753–60. doi:10.1177/1049732315617444

6.	Reilly MO, Parker N. “Unsatisfactory Saturation”: a critical exploration of the notion of saturated sample sizes in qualitative research. Qualitative Research. 2013;13(2):190–7. doi:10.1177/1468794112446106

7.	Braun V, Clarke V. Reflecting on reflexive thematic analysis. Qualitative Research in Sport, Exercise and Health. 2019;11(4):589–97. doi:10.1080/2159676X.2019.1628806

8.	Raja S, Rabinowitz EP, Gray MJ. Universal screening and trauma informed care: Current concerns and future directions. Families, Systems, & Health. 2021 Sep;39(3):526–34. doi:10.1037/fsh0000585

9.	Raja S, Hasnain M, Hoersch M, Gove-Yin S, Rajagopalan C. Trauma informed care in medicine: Current knowledge and future research directions. Family and Community Health. 2015;38(3):216–26. doi:10.1097/FCH.0000000000000071

10.	Ridout KK, Ridout SJ, Harris B, Felitti V. Universal Adverse Childhood Experience Screening in Primary Care. NEJM Catalyst. 2023 Feb 15;4(3). doi:10.1056/CAT.22.0106

11.	Küçükardalı-Cansever B, Lamson A, Sira N, Bridgland SA, Eagan S, Roberts E. Adverse Childhood Experiences (ACEs) Screening in Primary Care Settings for Adults: A Systematic Review. Journ Child Adol Trauma. 2025 Jun;18(2):377–93. doi:10.1007/s40653-025-00691-4

12.	Keen S, Lomeli-Rodriguez M, Joffe H. From Challenge to Opportunity: Virtual Qualitative Research During COVID-19 and Beyond. International Journal of Qualitative Methods. 2022 Apr;21:16094069221105075. doi:10.1177/16094069221105075
